# Progress in Medicine

**Published:** 1896-02-29

**Authors:** 


### Progress in Medicine.

### SUPRA-RENAL BODIES.

Gourfein[1] seems to have extracted from the supra-renal capsules a substance somewhat differing in character from that obtained by Schäfer and Oliver. Whereas the extract of the two latter investigators acted as a tonic to the vaso-motor system, raising the blood pressure, and when the vagus was divided increasing the force of cardiac action, the substance obtained by Gourfein exerted a depressing effect upon the heart and respiratory system of mammals, producing dyspnœa, which ended in death. The respiration ceased before the heart came to a standstill, but that failed even when artificial respiration was kept up. Vomiting also occurred while sufficient strength remained. The substance extracted by Schäfer and Oliver may possibly be, as suggested by them, an internal secretion; but the extract of Gourfein is obviously toxic in nature.

Various cases have been recorded in which the therapeutic effect of extract of supra-renal tissue has been tried in cases of Addison's disease. The most recent are by Sansom, Renger and Phear, and Murrell. In only one, that of Sansom,[2] can the result be called satisfactory. It was in a young man, aged 25, who had suffered from ill-health for about a year; and on coming under observation presented the signs and symptoms of Addison's disease. When treated with tabloids of supra-renal extract he gained fourteen pounds in weight during the space of a month. The two other cases referred to ended fatally. That recorded by Renger and Phear[3] was a woman, aged 28. She had suffered from symptoms of Addison's disease for two years. There was some improvement for four when taking tabloids of supra-renal extract. This improvement, however, was not maintained, and she sank and died. The third case, published by Murrell,[4] is still less satisfactory. A man, aged 30, who apparently had only suffered from impairment of health for three months before he came under observation, was given tabloids of supra-renal extract for three weeks; the symptoms became more marked during that time. The dose was increased, but still there was no improvement. Sheep's adrenals were then given, but vomiting occurred soon after eating them. They were changed for calves' supra-renals, but the same result followed, and the treatment was discontinued. The patient grew worse, and died two months after admission.

[1] Epitome, Brit. Med. Journal, Dec. 14, 1895. [2] Clinical Sketches, Nov., 1895. [3] Brit. Med. Journal, Jan. 18, 1896, p. 150. [4] Lancet, Feb. 1, 1896, p. 289.

